# Glucose patterns following alcohol and illicit drug use in young adults with type 1 diabetes: A flash glucose monitoring study

**DOI:** 10.1002/edm2.257

**Published:** 2021-05-06

**Authors:** Adam Pastor, Jennifer Conn, Margaret Loh, Casey L O'Brien, Jessie Teng, Sue Finch, Lisa Collins, Richard J MacIsaac, Yvonne Bonomo

**Affiliations:** ^1^ Department of Addiction Medicine St Vincent's Hospital Melbourne Fitzroy Vic. Australia; ^2^ Department of Medicine University of Melbourne Parkville Vic. Australia; ^3^ Department of Diabetes and Endocrinology Royal Melbourne Hospital Parkville Vic. Australia; ^4^ Department of Endocrinology and Diabetes St Vincent's Hospital Melbourne Fitzroy Vic. Australia; ^5^ Mental Health Services St Vincent's Hospital Melbourne Fitzroy Vic. Australia; ^6^ Department of Psychiatry University of Melbourne Parkville Vic. Australia; ^7^ School of Mathematics and Statistics University of Melbourne Parkville Vic. Australia

**Keywords:** alcohol drinking, diabetes, diabetes mellitus, harm reduction, health behaviour, interventions, substance‐related disorders, illicit drugs

## Abstract

**Introduction:**

To assess the effects of alcohol and illicit drug use in young adults (age 18–35) with type 1 diabetes (T1D) on flash glucose monitor sensor glucose (SG) readings.

**Methods:**

Twenty young adults with T1D were enrolled from a tertiary referral hospital outpatient department in Melbourne, Australia for a 6‐week prospective observational study using flash glucose monitoring (FGM). Glucometrics comparing substance using days (SUEDs) to those without substance use (non‐SUEDS) were analysed. The primary outcomes were the difference in mean SG values, its standard deviation and minutes/24‐h period out of range (SG <3.9 mmol/L or >10.0 mmol/L) between matched SUEDs vs non‐SUEDs. An interaction model with the primary effect of HbA1c on SG values was also performed.

**Results:**

There were no differences in the primary outcome measures between SUEDS and non‐SUEDs. However, there were differences in the regression coefficients for HbA1c and glucometrics between non‐SUEDs and SUEDs for mean SG, time out of range and time with SG > 10 mmol/L. This difference was also identified between non‐SUEDS and days of ≥40 g alcohol for mean SG.

**Conclusions:**

While there was no difference between glucometrics for SUEDs and non‐SUEDs on primary outcomes, HbA1C was found to be a less reliable predictor of glucose patterns in the 24‐h period following substance use than control days. Young adults with T1D need to monitor and respond to their glucose levels following substance use and engage in harm minimisation practices irrespective of baseline glucose control.

## Graphical abstract

Using FGM, we found no difference in glucometrics in young adults with type 1 diabetes following days of substance use than those without. However, HbA1C values were less predictive of glucometrics on days of substance use than those without substance use. Young adults with T1D, even those with adequate glucose control, need to engage in harm reduction measures, including closer monitoring of their glucose levels, should they choose to consume alcohol or illicit drugs.

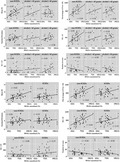

## 1 INTRODUCTION

Type 1 diabetes (T1D) is a chronic autoimmune condition of pancreatic β‐cells requiring lifelong administration of exogenous insulin and a commitment to a complex regimen of glucose monitoring, dietary choices and physical activity. Over 50% of individuals with T1D are diagnosed before the age of 18.^1^ This group faces specific challenges as they need not only to acquire the skills of diabetes self‐management but successfully navigate the transition from adolescence to young adulthood.

Among the developmental tasks that young people with T1D need to master is the ability to make safe choices regarding consumption of alcohol and other illicit drugs.^2^, ^3^ Although young adults with T1D use substances at similar rates to their peers,^4^, ^5^ they are at added risk of drug and alcohol‐related morbidity and mortality due to the potential interaction with their diabetes.^6^, ^7^

The pathways by which alcohol and illicit drug use interacts with T1D outcomes are multifactorial.^6^, ^7^ Notably, alcohol and illicit drugs can have direct metabolic effects on glucose levels. Alcohol can cause a delayed hypoglycaemia, the ‘morning after evening’ effect, which is frequently reported in clinical practice and been established in small case‐controlled studies.^8^, ^9^, ^10^ This delayed hypoglycaemia is secondary to the disruption of gluconeogenesis by alcohol, which, followed by a depletion of available glycogen stores overnight, and in the presence of exogenous insulin, precipitates hypoglycaemia. Previous efforts have been made to model appropriate insulin dosing responses to this physiologic mechanism^11^ but in practice this approach is often limited by the lack of continuous glucose monitoring required to make fine adjustments to insulin dosing. Amphetamine‐type stimulants and cocaine predispose to hyperglycaemia and diabetic ketoacidosis through their adrenergic effects.^12^, ^13^ Recent studies have also shown an increased rate of presentations for diabetic ketoacidosis in jurisdictions that have legalized cannabis.^12^ Further impacts are likely in real world environments, where the effects of intoxication can impair monitoring and responding to glucose levels. Furthermore, the impact of substances on mental health can lead to decreased motivation for diabetes self‐management and increased levels of diabetes‐related distress.

Due to the interaction between substance use and glucose control in TID, an understanding of the magnitude, direction and predictability of this interaction is critical for enhancing strategies for harm reduction. Studies to date have been either tightly controlled in a laboratory environment^10^ or of short duration, typically 3–5 days, increasing the risk of observer effects.^14^ To date, they have only attempted to study and record alcohol consumption and there have been no prospective clinical studies exploring the effect of a variety of other recreational drugs on glucose levels in a naturalistic environment. This absence of prospective experimental evidence on drinking and illicit drugs has led to inconsistent harm minimisation messages.^15^

New diabetes technologies provide novel ways of exploring interactions between alcohol and drug use and glucose levels. One such technology is flash glucose monitoring (FGM), which involves the use of a factory‐calibrated sensor inserted into subcutaneous tissue. The sensor measures prevailing interstitial glucose levels at frequent intervals and the results are displayed on a reader which scans the sensor. FGM provides a 24‐h glucose profile and reduces the need for finger pricks.^16^ Randomized trials and observational studies have confirmed the acceptability of FGM in adults with T1D, as well as its effectiveness in optimising glucose parameters such as reducing time in hypoglycaemia.^16^, ^17^, ^18^

To date, FGM has not been used as a research tool to document glucose levels following the use of alcohol and illicit drugs. The aim of this study was to use FGM as a novel method of documenting the interaction between substance consumption and metabolic control and to establish the specific effects of substance use on glucose metrics in a real‐world environment in young adults with T1D.

## 2 MATERIALS AND METHODS

### 2.1 Study design and participant selection

Participants in this six‐week prospective observational study were recruited from an outpatient adult T1D clinic at St Vincent's Hospital, Melbourne. They were aged 18–35 years and had been living with T1D for >1 year. Participants were identified by a diabetes educator and consented for the study by the first author. Inclusion criteria were consumption of ≥40 g of alcohol (typically three to four 375 ml bottles of beer or half a bottle of wine) on a single occasion and/or had taken an illicit drug in the previous six weeks with stated intention to do so again in the coming 6 weeks. Exclusion criteria included high risk substance use, defined as a World Health Organisation ASSIST^19^ score ≥ 27, or treatment for an alcohol or drug use disorder in the previous 6 months. Also excluded were people with high baseline levels of psychological distress, defined as a Kessler Psychological Distress Scale (K10) score of >20^20^ or a known diagnosis of schizophrenia or psychotic disorder not related to drug use. Participants with an admission for diabetic ketoacidosis within the previous month or a Gold hypoglycaemia score of ≥4 were also excluded.^21^ Females were excluded if they were pregnant or planning pregnancy. Participants with unstable or severe renal, cardiac, respiratory or liver disease were also excluded. An addiction medicine nurse performed a 15‐min episode of counselling prior to trial registration highlighting the risks of substance use. Participants were given the option to reduce their alcohol or drug use and not participate in the study. The study was approved by the St Vincent's Hospital Melbourne Human Research Ethics Committee (HREC/16/SVHM/253). All participants provided written informed consent.

A sample size of 17 was calculated using Minitab 19, based on an expected difference between the two groups in sensor glucose (SG) time outside of target range of 30 min in 24 h, assumed standard deviation 30, with confidence level of 95% and a power of 0.8. Allowing for an attrition rate of 20%, the study aimed to recruit up to 24 participants.

### 2.2 Procedures

Participants were enrolled between April 2017 and January 2019. At study enrolment, demographics, medical history, drug and alcohol history, physical examination and an HbA1c from within three months of recruitment were recorded, along with regular medications and scores on Gold hypoglycaemia, K10, WHO ASSIST and Problem Areas in Diabetes (PAID) questionnaires.^22^ Females performed a urine βHCG and agreed to maintain effective contraception during the trial.

Upon enrolment, participants were supplied with a FGM reader and three glucose sensors (Abbott Freestyle^®^ Libre). As each sensor lasts 14 days, this provided six weeks of FGM data. The sensors were applied and used as per the product license in Australia with assistance from a physician or diabetes educator. Participants were asked to keep an activity diary to record daily alcohol and drug use. Any changes to regular routine, such as sick days, changes in diet, travel or vigorous exercise were recorded. It was also noted whether the day was a work or weekend day. Participants were reviewed in person fortnightly for collection of activity diaries, SG data and information regarding adverse events. Participants continued regular diabetes management and were provided with contact numbers for support.

### 2.3 Matching

Each participant diary was reviewed and substance using days (SUED) were identified as those involving alcohol, cannabis, stimulants (cocaine, ecstasy, methamphetamine or other) or polysubstance (three or more illicit drugs). The amount of alcohol consumed was also recorded. Each SUED was assigned a matched non‐substance‐using (non‐SUED) day by two researchers blinded to the SG data. The matching day was assigned by reviewing the activity diary of each participant and selecting a comparable day within the six‐week period not affected by substance use. This day typically matched the day of the week of the SUED, was not during the first two weeks of monitoring and excluded atypical exercise or meals. This design enabled each participant to act as their own control. The SG data analysed were from the 24‐h period from the commencement of substance use compared with 24‐h period from the matched control day.

### 2.4 Statistical analysis

**FIGURE 1:**
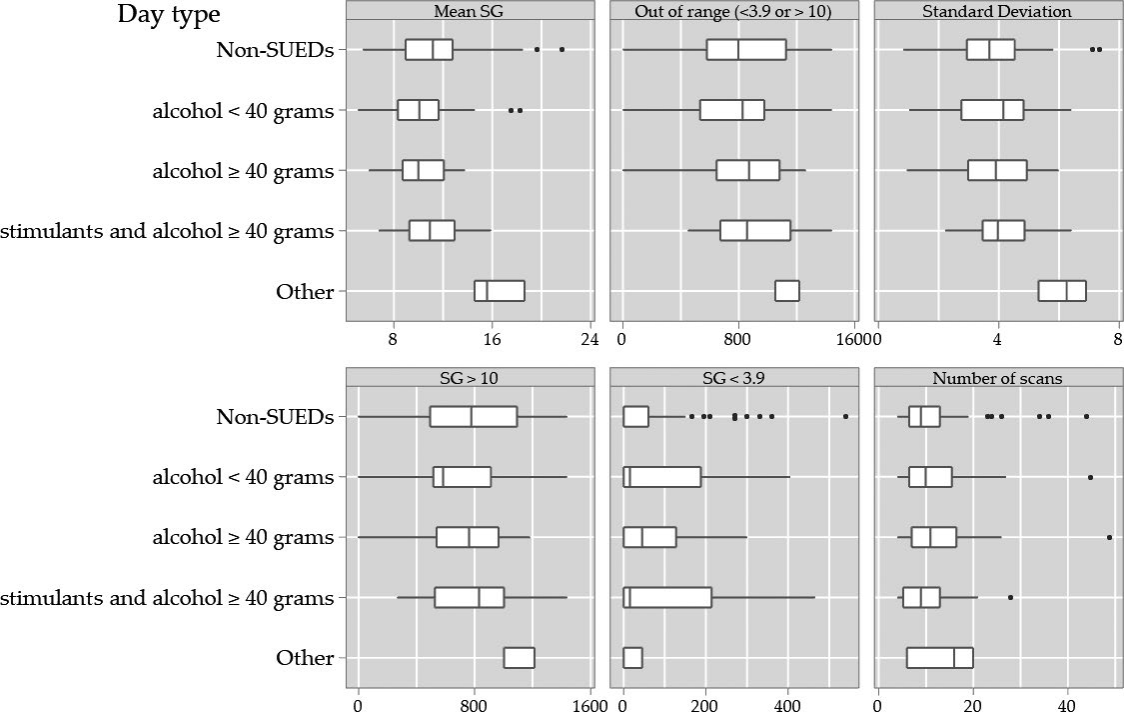
Boxplots of glucose outcome measures by type of substance use. # Other includes 2 episodes cannabis use and 3 episodes cannabis and stimulant use

We analysed each 24‐h period using the glucometrics recommended by the Juvenile Diabetes Research Foundation Artificial Pancreas Project Consortium^23^ to compare SUEDs and non‐SUEDs. Alcohol use was divided into two groups according to intake of <or ≥40 g. As stimulants were always consumed with ≥40 g of alcohol, this combination formed a separate group. The primary outcomes were the difference in mean SG level, standard deviation and minutes/24 h period out of range (SG < 3.9 mmol/L or >10.0 mmol/L). Secondary measures included mean differences in SG < 3.9 mmol/L, SG > 10 mmol/L, and mean number of FGM scans (Figure 1). For statistical analysis, transformation of two outcomes was required: a log transformation with a 10‐point location shift was applied for minutes/24 h of SG < 3.9 mmol/L, and an inverse square root to the number of FGM reader scans.

For each outcome, two types of general linear mixed models were fitted. A main effects model considered SUEDs as the primary explanatory variable of interest, but included age, gender and HbA1c as covariates. In the interaction model, the primary effect of interest was the interaction of SUEDs and HbA1c but included age and gender. The test statistics reported are for the primary effects of interest in each case. All models included participant and day pair as random effects. For the main effects model, the difference of means is provided; this is the difference in the mean outcome comparing non‐SUEDs and SUEDs. For the interaction model, the difference of “slopes” (regression coefficients for HbA1c) according to non‐SUEDs or SUEDs is provided.

Both the main effects and interaction models were applied to non‐SUEDs and all SUEDs. These models were then applied to analyse alcohol consumption effects alone comparing non‐SUEDs, days after <40 g alcohol were consumed, and days ≥40 g alcohol were consumed. Days where any other drugs were consumed were excluded. (Table 2) Due to the small numbers in the stimulant and ≥40 g alcohol consuming group, for analyses considering this group, only a main effects model is reported. The results reported are from five statistical models (the primary and secondary measures) reported for each outcome for the three groups of SUEDs. The results for the first two groupings of SUEDs are reported in Table 2. Models for the third grouping are included in Appendix 1.

## 3 RESULTS

A cohort of 20 participants was recruited with two withdrawing prior to completion (one lost to follow up and one hospitalized with diabetic ketoacidosis during the 6‐week period). In both participant withdrawals, sufficient data had been collected to analyse at least one substance using event. The demographic and clinical characteristics of participants are described in Table 1.

**TABLE 1:** Study participants (*n* = 20)

Demographics	Median (IQR)	Diabetes‐related measures at baseline	Median (IQR)
Age	29 (24,30)	Age of diagnosis (years)	10 (4,20)
	*n* (%)	Years since diagnosis	20 (4,23)
Female	14 (70%)	HbA1C mmol/mol (%)	68.3 (8.4) (55.2 (7.2), 79.2 (9.4))
Never Married	17 (85%)	Total daily dose insulin (units)	44 (36,66)
Born in Australia	15 (75%)	PAID score	15 (7.5, 28.75)
English first language	19 (95%)		*n* (%)
Employed or Studying	19 (95%)	CSII^a^	4 (20%)
		Severe diabetes distress (PAID >40)	4 (20%)
Substance use baseline (6 weeks prior to study)		Substance use during study period (6 weeks of study)	
Alcohol	20 (100%)	Alcohol	20 (100%)
Cannabis	8 (40%)	Cannabis	4 (20%)
Stimulants	7 (35%)	Stimulants	7 (35%)
Poly‐substance	4 (20%)	Polysubstance	4 (20%)

^a^ Continuous subcutaneous insulin infusion.

There were no mean differences detected in the primary outcome measures between SUEDS and non‐SUEDs for any substance group. Raw numerical data comparing glucometrics between non‐SUEDs (*n* = 61) and SUED days for alcohol <40 g (*n* = 17), alcohol ≥40 g (*n* = 31) and stimulants and ≥40 g alcohol (*n* = 8) are shown in Figure 1 and the main effects and interaction models are in Tables 2 and 3.

**TABLE 2:** Main effects and Interaction models for Non‐SUEDs – all SUEDs

Outcome	Test statistic	*P*‐value		
Mean SG (mmol/L)			Difference of coefficients	95% CI
Main effects	*F*_(1,101) =1_.04	.309		
Interaction	*F*_(1,59)_ = 8.12	.006*	0.75	0.22, 1.27
Out of range (min/24 h)			Difference of coefficients	95% CI
Main effects	*F*_(1,99)_ = 0.05	.832		
Interaction	*F*_(1,99)_ = 4.47	.037*	60.55	3.74, 117.37
Standard deviation			Mean difference	95% CI
Main effects	*F*_(1,101)_ = 2.91	.091	−0.31	−0.66, 0.05
Interaction	*F*_(1,100)_ = 0.43	.515		
SG > 10 (min/24 h)			Difference of coefficients	95% CI
Main effects	*F*_(1,99)_ = 0.13	.714		
Interaction	*F*_(1,99)_ = 5.58	.020*	77.09	12.36, 141.82
Log (SG < 3.9 + 10)			Mean Difference	95% CI
Main effects	*F*_(1,101)_ = 2.36	.128	−0.35	−0.81, 0.10
Interaction	*F*_(1,100)_ = 1.93	.168		
Inverse square root # of scans			Mean Difference	95% CI
Main effects	*F*_(1,60)_ = 0.36	.553	0.01	−0.01, 0.02
Interaction	*F*_(1,59)_ = 0.12	.730		

* *P* < 0.05.

**TABLE 3:** Main effects and Interaction models for non‐SUEDs – <40 g alcohol – ≥40 g alcohol

Outcome	Test statistic	*P*‐value		
Mean SG (mmol/L)			Difference of coefficients	95% CI
Main effects	*F*_(2.60)_ = 1.19	.312		
Interaction	*F*_(2,58)_ = 2.68	.077		
Non‐SUEDs – (<40 g)			0.49	−0.30, 1.28
Non‐SUEDs – (≥40 g)			0.83	0.06, 1.61*
(<40 g)–(≥40 g)			0.34	−0.64, 1.33
Out of range (min/24 h)			Difference of coefficients	95% CI
Main effects	*F*_(2,73)_ = 0.21	.811		
Interaction	*F*_(2,73)_ = 1.38	.257		
Non‐SUEDs – (<40 g)			0.81	−0.54, 2.15
Non‐SUEDs – (≥40 g)			0.65	−0.43, 1.73
(<40 g)–(≥40 g)			−0.15	−1.67, 1.37
Standard deviation			Mean difference	95% CI
Main effects	*F*_(2,73)_ = 0.41	.665		
Interaction	*F*_(2,73)_ = 0.46	.631		
Non‐SUEDs – (<40 g)			0.01	−0.57, 0.59
Non‐SUEDs – (≥40 g)			−0.21	−0.68, 0.27
(<40 g)–(≥40 g)			−0.21	−0.83, 0.42
SG >10 (min/24 h)			Difference of coefficients	95% CI
Main effects	*F*_(2,74)_ = 0.54	.586		
Interaction	*F*_(2,73)_ = 1.85	.164		
Non‐SUEDs – (<40 g)			0.01	−0.57, 0.59
Non‐SUEDs – (≥40 g)			−0.21	−0.68, 0.27
(<40 g)–(≥40 g)			−0.21	−0.83, 0.42
Log(SG <3.9 + 10)			Mean difference	95% CI
Main effects	*F*_(2,76)_ = 2.36	.166		
Interaction	*F*_(2,77)_ = 1.93	0.207		
Non‐SUEDs – (<40 g)			−0.40	−1.14, 0.34
Non‐SUEDs – (≥40 g)			−0.56	−0.05, 1.17
(<40 g)–(≥40 g)			−0.16	−0.95, 0.64
Inverse square root # of scans			Mean difference	95% CI
Main effects	*F*_(2,72)_ = 0.82	0.444		
Interaction	*F*_(2,71)_ = 0.34	0.714		
Non‐SUEDs – (<40 g)			0.01	−0.02, 0.03
Non‐SUEDs – (≥40 g)			−0.01	−0.03, 0.01
(<40 g)–(≥40 g)			−0.02	−0.04, 0.02

* *P* < 0.05.

**FIGURE 2:**
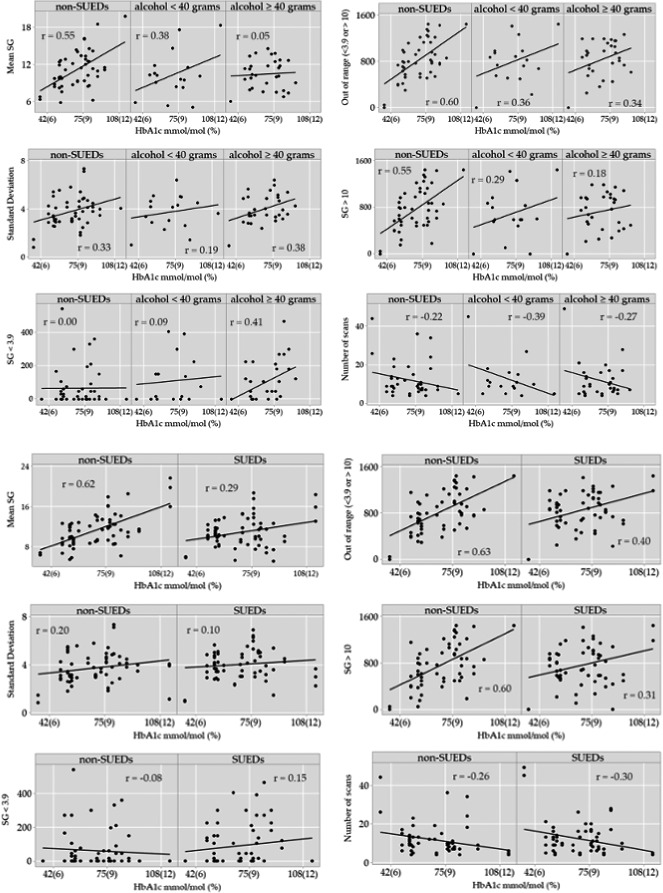
Relationships of Hba1c to glucose outcome measures for non‐SUEDs–all SUEDS and non‐SUEDS–alcohol consuming days (Pearson correlations are provided in each panel)

Statistically significant differences were observed between SUEDs and non‐SUEDS in the relationship between HbA1c and mean SG, time out of range (min/24 h) and time with SG > 10 mmol/L (min/24 h). A significant difference was also detected in this relationship on days when ≥40 g of alcohol was used (compared with non‐SUEDs) for mean SG values but not for other outcome measures. The above relationships are represented graphically in Figure 2 with the Pearson coefficient (*r*) representing the strength of the relationship between HbA1c and the outcome. As shown in the Tables 2 and 3, and Figure 2, the relationship between HbA1c and glucometrics was weaker on SUEDs than non‐SUEDs.

## 4 DISCUSSION

This study is the first to record glucose outcomes following alcohol and drug use in a naturalistic context over a 6‐week period using FGM. We did not find a direct relationship between glucose parameters and substance use. However, our data showed a weakening of the relationship between HbA1c and a range of glucose parameters on SUEDs versus non‐SUEDs. In most studies from the literature, a linear relationship exists between HbA1c and glycaemic control parameters such as mean SG and time out of range.^24^, ^25^ We found that HbA1c was less closely associated with mean SG, time out of range (SG < 3.9 and >10 mmol/L) and time with SG > 10 mmol/L on days when substances were used. Also, a dose‐dependent effect was observed for those days when alcohol alone was consumed. Regardless of baseline HbA1c, participants had less predictable glucose levels on the days when they consumed ≥40 g of alcohol, than when they consumed <40 g of alcohol or when no alcohol was consumed.

We did not detect a difference in hypoglycaemia rates between SUEDs and non‐SUEDs. Previous studies performed in clinical laboratory settings with standardized doses of alcohol being administered have generally reported alcohol‐induced hypoglycaemia. This is true for studies performed in UK, Germany and Italy. In contrast, those studies performed in naturalistic settings across different countries have yielded variable results^9^, ^15^ typically not measuring a change in mean glucose but finding an increase in glucose variability. Our study findings therefore concur with the literature and suggest that a threshold level of alcohol consumption is relevant.^9^, ^15^

Our study did not establish an impact on SG when stimulants were used concurrently with alcohol consumption. However, the number of events available for analysis in our sample was small (*n* = 8), limiting any definite conclusions. Interpretation of effects of cannabis on SG was not possible because of small numbers of cannabis users (*n* = 4). While they are no comparable studies including illicit drug, these results would be expected to vary internationally as Australia has a larger prevalence of methamphetamine users (and correspondingly lower numbers of cocaine users) than Europe, UK or USA. Trends to legalize cannabis in North and South America are also likely to impact.

This is the largest, and to date the longest, naturalistic study of the effect of alcohol and illicit drugs on glucose levels in young adults with T1D. We identified that, regardless of baseline glycaemic control, glucose levels are less predictable on days when substances are used. This suggests that young adults with T1D need to be provided with health promotion messages that highlight this information and be encouraged to intensify glucose monitoring when drinking alcohol and/or using illicit drugs irrespective of baseline glycaemic control. Larger real‐world studies, particularly on currently illicit drugs, are required as the patterns of substance use change both in Australia and internationally. Research is also required to clarify the most effective health messages for this cohort, as well as the role of closed loop insulin delivery systems and FGM to facilitate harm reduction.

### 4.1 Limitations

The naturalistic setting provides this study with both strengths and weaknesses. It more closely reflects the social context in which substances are consumed and, thus, the outcomes are likely to be more clinically relevant for young adults with diabetes. However, outside of a laboratory environment, it is difficult to control for wider variables likely to affect glucose parameters and this study may, thus, have underestimated the direct metabolic effects of substance use. As an example, as stimulants are rarely used without alcohol in real‐world settings, differences related to stimulant use only may not have been detected. Furthermore, as participants were selecting their own beverages, we could not analyse the effects of different alcohol types (eg. white wine vs red wine vs beer) as these were frequently mixed. The metabolic effect of different alcohol types on BGLs may be important.

Considering the stigma associated with substance use, recruitment for studies of this kind is challenging. This is particularly so in a clinical environment where participants may be reluctant to admit to illicit drug use so as not to be seen to disappoint their treatment team. While the largest study of this type and most diverse in terms of substances documented, only 20 participants were recruited from a single tertiary referral centre with a largely urban Australian population. A larger study with more substance using event days may potentially have allowed detection of effects consistent with those found in laboratory studies. Subgroups such as those with substance use disorders, and severe psychiatric and medical comorbidities were also excluded. Participants in our sample consumed more alcohol than cannabis or stimulants and there were no opioid users. Activity diaries may have been subject to recall bias. While interviews were performed with explicit reassurance of confidentiality and results were not shared with treating clinicians, there remains the possibility of under‐reporting. Reliable estimates of quantity were possible for alcohol but not for illicit drugs. Finally, the group using stimulants were also using alcohol simultaneously, which may have modified the metabolic effect resulting in less hyperglycaemia than might otherwise have been anticipated.

Despite attempts to minimize any changes in substance use patterns from baseline, an observer effect may have resulted in more or less frequent substance use, used in a more or less harmful manner. As SG was available to the participants in real time, participants may have responded by altering their insulin doses or diet minimising the metabolic effect of substance use. While reflecting real world conditions, this is likely to have resulted in an underestimating of the metabolic effect of alcohol and illicit drugs. Notwithstanding the limitations, the findings provide insights into alcohol and drug use and it impacts in young adults with T1D.

## 5 CONCLUSION

Using FGM, we found that HbA1C values in young adults with T1D was less predictive of glucometrics on days of substance use than those without substance use. As alcohol and illicit drug use are common in this age group, clinicians need to be aware of the potential impact of substance use on glucose parameters in young adults with T1D. Even those young adults with adequate glucose control need to engage in harm reduction measures, including closer monitoring of their glucose levels, should they choose to consume alcohol or illicit drugs.

## CONFLICT OF INTEREST

Richard MacIsaac has received research grants from Novo Nordisk, Servier, Medtronic, The Rebecca Cooper Medical Research Foundation, St Vincent's Research Foundation, The Juvenile Diabetes Research Foundation, Grey Innovations, The Diabetes Australia Research Trust/Program and The National Health and Medical Research Council of Australia. He has also has received honoraria for lectures from Eli Lilly, Novo Nordisk, Sanofi Aventis, Astra Zeneca, Merck Sharp & Dohme, Novartis and Boehringer Ingelheim and is on the advisory boards for Novo Nordisk, Boehringer Ingelheim‐Eli Lilly Diabetes Alliance and Astra Zeneca. Travel support has been supplied by Novo Nordisk, Sanofi and Boehringer Ingelheim. He has been a principal investigator for industry sponsored clinical trials run by Novo Nordisk, Bayer, Johnson‐Cilag and Abbvie. All other authors declare no conflicts of interest.

## AUTHOR CONTRIBUTION

AP conceptualized the study, collected data, wrote the manuscript, acquired funding. JC assisted in conceptualizing the study, supervision, reviewing original draft and reviewing and editing the final draft of the manuscript. ML recruited the participants, assisted in providing resources, developing study methodology and reviewing the manuscript. CLO assisted with conceptualisation, data curation, reviewing the manuscript. JT assisted with resources, validation of data, conceptualization and study methodology. SF lead the formal statistical analysis, reviewed the manuscript and assisted with trial methodology. LC assisted with data curation, data collection, project administration and reviewing the first and final draft of manuscript. RJM and YB were involved in trial conceptualization, methodology, supervision and reviewing and editing the first and final draft of the manuscript.

## Data Availability

The data that support the findings of this study are available on request from the corresponding author. The data are not publicly available due to privacy or ethical restrictions.

## APPENDIX 1

This appendix provides the analyses for the third operationalisation of SUDS: no substance use, <40 g of alcohol (only), 40 g of alcohol or more (only), 40 g of alcohol or more and other stimulant use. This analysis focussed on the effects of stimulants, and so excluded days on which participants took: cannabis only, cannabis and stimulants without alcohol, or cannabis and alcohol.

**Table:**

Outcome	Test statistic	*P*‐value		SUDs: No Substances, <40 g EtOH, 40+ g EtOH, 40+ g EtOH & stimulants	
Model					
Mean BGL				Mean difference	95% CI
Main effects	*F*_(3,83)_ = 0.79	.501	0 g–<40 g	0.84	−0.49, 2.16
			0 g–40 g+	0.60	−0.46, 1.66
			0 g–40 g+ & stimulants	0.55	−1.33, 2.43
			<40 g–40 g+	−0.23	−1.70, 1.24
			<40 g–40 g+ & stimulants	−0.29	−2.45, 1.87
			40 g+–40 g+ & stimulants	−0.06	−2.06, 1.95
Out of range			Mean difference	95% CI	
Main effects	*F*_(3,91)_ = 0.17	.915	0 g–<40 g	34.76	−111.00, 180.53
			0 g–40 g+	−21.08	−138.66, 96.50
			0 g–40 g+ & stimulants	−18.45	−224.40, 187.51
			<40 g–40 g+	−55.84	−215.15, 103.47
			<40 g–40 g+ & stimulants	−53.21	−288.07, 181.65
			40 g+–40 g+ & stimulants	2.63	−215.56, 220.83
Standard deviation				Mean difference	95% CI
Main effects	*F*_(3,92)_ = 0.44	.728	0 g–<40 g	−0.10	−0.67, 0.46
			0 g–40 g+	−0.22	−0.68, 0.24
			0 g–40 g+ & stimulants	−0.33	−1.13, 0.48
			<40 g–40 g+	−0.11	−0.74, 0.51
			<40 g–40 g+ & stimulants	−0.22	−1.14, 0.70
			40 g+–40 g+ & stimulants	−0.11	−0.96, 0.75
BGL >10				Mean difference	95% CI
Main effects	*F*_(3,93)_ = 0.35	.788	0 g–<40 g	81.47	−83.22, 246.16
			0 g–40 g+	16.25	−116.61, 149.12
			0 g–40 g+ & stimulants	−18.61	−250.98, 213.76
			<40 g–40 g+	−65.22	−245.16, 114.73
			<40 g–40 g+ & stimulants	−100.08	−364.99, 164.84
			40 g+–40 g+ & stimulants	−34.86	−280.95, 211.23
BGL <3.9 transformed				Mean difference	95% CI
Main effects	*F*_(3,96)_ = 2.26	.086	0 g–<40 g	−0.48	−1.18, 0.22
			0 g–40 g+	−0.59	−1.16, −0.03
			0 g–40 g+ & stimulants	0.41	−0.57, 1.39
			<40 g–40 g+	−0.11	−0.88, 0.65
			<40 g–40 g+ & stimulants	0.89	−0.22, 2.00
			40 g+–40 g+ & stimulants	1.00	−0.03, 2.04
Number of scans transformed				Mean difference	95% CI
Main effects	*F*_(3,90)_ = 1.56	.205	0 g–<40 g	0.01	−0.01, 0.04
			0 g–40 g+	−0.01	−0.03, 0.02
			0 g–40 g+ & stimulants	0.03	−0.01, 0.07
			<40 g–40 g+	−0.02	−0.05, 0.01
			<40 g–40 g+ & stimulants	0.02	−0.03, 0.06
			40 g+–40 g+ & stimulants	0.04	−0.00, 0.08
